# Associations between dietary fatty acid and plasma fatty acid composition in non-alcoholic fatty liver disease: secondary analysis from a randomised trial with a hypoenergetic low-carbohydrate high-fat and intermittent fasting diet

**DOI:** 10.1017/S0007114524001673

**Published:** 2024-08-28

**Authors:** Veronika Tillander, Magnus Holmer, Hannes Hagström, Sven Petersson, Torkel B. Brismar, Per Stål, Catarina Lindqvist

**Affiliations:** 1 Division of Clinical Chemistry, Cardio Metabolic Unit, Department of Laboratory Medicine, Karolinska Institutet, Stockholm, Sweden; 2 Unit of Gastroenterology and Hepatology, Department of Medicine Huddinge, Karolinska Institutet, Stockholm, Sweden; 3 Division of Hepatology, Department of Upper GI, Karolinska University Hospital, Stockholm, Sweden; 4 Department of Clinical Science, Intervention and Technology, Division of Medical Imaging and Technology, Karolinska Institutet, Stockholm, Sweden; 5 Department of Medical Radiation Physics and Nuclear Medicine, Karolinska University Hospital, Stockholm, Sweden; 6 Department of Radiology, Karolinska University Hospital in Huddinge, Stockholm sE-14186, Sweden; 7 Medical Unit Clinical Nutrition, Karolinska University Hospital, Stockholm, Sweden

**Keywords:** Non-alcoholic fatty liver disease, Metabolic dysfunction-associated steatotic liver disease, Steatotic liver disease, Diet intervention, Plasma fatty acid composition

## Abstract

Dietary fatty acids (FA) affect metabolic risk factors. The aim of this study was to explore if changes in dietary fat intake during energy restriction were associated with plasma FA composition. The study also investigated if these changes were associated with changes in liver fat, liver stiffness and plasma lipids among persons with non-alcoholic fatty liver disease. Dietary and plasma FA were investigated in patients with non-alcoholic fatty liver disease (*n* 48) previously enrolled in a 12-week-long open-label randomised controlled trial comparing two energy-restricted diets: a low-carbohydrate high-fat diet and intermittent fasting diet (5:2), to a control group. Self-reported 3 d food diaries were used for FA intake, and plasma FA composition was analysed using GC. Liver fat content and stiffness were measured by MRI and transient elastography. Changes in intake of total FA (*r* 0·41; *P* = 0·005), SFA (*r* 0·38; *P* = 0·011) and MUFA (*r* 0·42; *P* = 0·004) were associated with changes in liver stiffness. Changes in plasma SFA (*r* 0·32; *P* = 0·032) and C16 : 1*n*-7 (*r* 0·33; *P* = 0·028) were positively associated with changes in liver fat, while total *n*-6 PUFA (*r* −0·33; *P* = 0·028) and C20 : 4*n*-6 (*r* −0·42; *P* = 0·005) were inversely associated. Changes in dietary SFA, MUFA, cholesterol and C20:4 were positively associated with plasma total cholesterol and LDL-cholesterol. Modifying the composition of dietary fats during dietary interventions causes changes in the plasma FA profile in patients with non-alcoholic fatty liver disease. These changes are associated with changes in liver fat, stiffness, plasma cholesterol and TAG. Replacing SFA with PUFA may improve metabolic parameters in non-alcoholic fatty liver disease patients during weight loss treatment.

Non-alcoholic fatty liver disease (NAFLD) has increased steadily, and the estimated global prevalence is reaching around 38 % in adults^(1)^. NAFLD (recently proposed to be termed as ‘metabolic dysfunction-associated steatotic liver disease’ although this paper keeps the term NAFLD to align with the inclusion and exclusion criteria in the original study) is a potentially progressive disease with the risk for advanced liver complications such as cirrhosis or hepatocellular carcinoma^(2)^. The future burden of NAFLD on healthcare systems around the world will become a challenge. In addition, NAFLD has been suggested to be an independent risk factor for atherosclerotic CVD, even after adjusting for other CVD risks related to metabolic disorders^(3,4)^. There is a lack of effective pharmacological treatment for NAFLD. The most effective therapy to reduce steatosis, and complications thereof, is weight reduction through lifestyle changes, including energy restriction. The current recommendation is to promote a weight loss of 5–10 %, usually by hypoenergetic diets^(5)^.

Dietary patterns rich in trans-SFA and SFA and diets with a high intake of fast and simple carbohydrates are important risk factors for CVD^(6)^. Therefore, in addition to the reduction of total energy intake, the ‘Mediterranean diet’ is recommended for patients with NAFLD, due to the diet’s risk-reducing effects on CVD^(7)^.

The liver is an essential organ for lipid modulation and has the capacity for *de novo lipogenesis* (DNL) and further elongation and desaturation of SFA to both MUFA and certain PUFA. The majority of lipids that accumulate during steatosis originate from plasma FA released during lipolysis in the adipose tissue. However, one-third of the accumulating lipids during steatosis originate directly from the diet or hepatic DNL, where the latter is known to increase in patients with obesity and NAFLD^(8)^. Substrate-to-product ratios of certain enzymes (such as stearoyl-CoA desaturase (SCD1) and δ5-desaturase (D5D)) can be viewed as a crude measurement of the adaption by hepatic lipid metabolism to changes in total energy intake and/or in incoming dietary FA. Since SCD1 is the rate-limiting step in the desaturation of SFA (in particular C16:0 and C18:0) to their respective MUFA, the plasma SCD1 index has been used as a rough measurement of hepatic lipid metabolism to handle high amounts of incoming FA (dietary or from lipolysis) as well as FA produced by increased rate of hepatic DNL^(9–11)^. The D5D index could be viewed as a rough indicator of the liver’s capacity to supply the body with endogenously synthesised PUFA, such as C20 : 4*n*-6^(9,12,13)^. Dietary lipids can influence the tissue composition of FA, not only by being a direct source of FA for incorporation into membrane lipids but also by affecting the regulation of hepatic lipid metabolism. Observational and population-based studies have shown that diets rich in PUFA increase the plasma content of PUFA and that high diet content of PUFA, as well as increased plasma content thereof, correlates with a lower amount of liver fat even after adjustments of weight or other relevant anthropometrical values^(14–18)^. However, few studies have explored how dietary changes in FA intake during hyperenergetic conditions affect plasma composition in relation to liver parameters in patients with NAFLD.

We previously showed that two different hypoenergetic dietary interventions, one using an intermittent fasting approach (5:2 diet) and one using a low-carbohydrate high-fat diet (LCHF diet), were superior in reducing liver fat in patients with NAFLD compared with a standard of care (SoC) control group in a 12-week intervention^(19)^. In this *post hoc* analysis, we investigate if differences in dietary fat composition during the study generated differences in the plasma configuration of FA. Furthermore, the study aimed to investigate if these changes are associated with changes in liver fat, liver stiffness and plasma lipids among persons with NAFLD.

## Methods

### Study participants

A detailed description of the primary study protocol and outcomes has been reported by Holmer *et al.* ^(19)^. In short, the primary aim of the open-label randomised controlled trial was to investigate the effect of a hypoenergetic LCHF diet and hypoenergetic intermittent fasting (5:2) on liver fat reduction, compared with an SoC, in patients with NAFLD. Patients (*n* 74) with clinically confirmed NAFLD were recruited at the Department of Hepatology, Karolinska University Hospital, Stockholm, between September 2017 to April 2020. Participants were randomised in a 1:1:1 ratio to a 12-week diet treatment with either LCHF, 5:2 or SoC. The study was approved by the Regional Ethics Committee in Stockholm, Sweden (in accordance with the ethical standards of the 1964 Declaration of Helsinki and its later amendments), and registered at Clinicaltrials.gov (NCT03118310). All participants provided written consent to be involved in the study. Out of the sixty-four participants who completed the study, food records and plasma samples for fatty acid (FA) analysis from the baseline and end point of the study were available for forty-eight of them. Two individuals had incomplete liver MRI scans, and one had incomplete measurements for BMI (kg/m^2^) calculations, and thus, they were excluded from further analysis of liver and blood parameters. For an outline of the participants in this *post hoc* analysis, see online Supplementary Fig. S1.

### Diets and food records

Participants provided self-reported food intake information by completing food diaries for three consecutive days during the week preceding the randomisation phase and again during the last week before the conclusion of the study at 12 weeks. The participants were asked to include at least 1 d of the weekend in the 3 d food diaries. The data were converted into energy percentages of total energy intake (E%) for FA and as cholesterol density (in g/1000 kcal). The Dietist Net Pro software (Kost och Näringsdata) was employed, relying on food composition tables from the Swedish National Food Administration^(19,20)^. To account for the differences in energy intake during fasting and non-fasting days for the participants randomised to the 5:2 diet, the self-reported intake was weighted by a factor of 2 or 5, respectively, and further reported as daily mean intake.

At baseline, the LCHF and 5:2 groups received oral instructions and diet-specific materials (including recipes for meals) from a specialist dietitian. In addition, participants received nutritional counselling and follow-up phone calls at week 2, 4 and 8 as well as a visit at week 6. The recipes provided for the two groups were based on Swedish food traditions and adjusted to fit the macronutrient composition and energy intake planned for each intervention. For a detailed description of the information that was given to each of the study groups, see Holmer *et al.* ^(19,20)^.

The LCHF diet had a macronutrient composition of 5–10 E% carbohydrates, 15–40 % protein and 50–80 E% of fat. The diet was based on a high intake of animal protein sources, (such as fish, eggs, meat and dairy products such as cream, butter and cheese), surface vegetables and vegetable oils. The intake of carbohydrate-rich products (such as bread, pasta, rice, potatoes, carbohydrate-rich fruits, sugar drinks and juices) was markedly reduced. The planned average daily energy intake was 1600 kcal for women and 1900 kcal for men and was shown to be followed according to the reported food intake^(20)^.

The daily mean energy intake for the participants of the 5:2 group was planned to be the same as for the LCHF diet (1600 kcal for women and 1900 kcal for men). This was managed by instructions to the participants to reduce their daily energy intake to 500 kcal for women and 600 kcal for men during two non-consecutive fasting days per week free of choice. Here the participants had energy-calculated recipes to choose from for their three main meals each fasting day. The remaining 5 d of the week had meals planned to comprise 2000 kcal/d for women and 2400 kcal/d for men, with a macronutrient composition of 48 E% of carbohydrates, 20 E% of protein and 32 E% of fat, according to the Nordic Nutrition Recommendations 2012^(21)^.

Participants in the SoC group received consultation by a physician in the beginning of the study. The participants were advised to follow a healthy diet, meaning a low intake of sugar-rich items, an increased intake of dietary PUFA and MUFA and a reduced consumption of food enriched in SFA. They were also instructed to have regular meals and to avoid large serving sizes. All participants, irrespective of diet group, were told not to change their daily/weekly physical activity during the study and to minimise their alcohol consumption.

### Fatty acid composition of total lipids in plasma

Plasma lipids of fasting blood samples, collected in EDTA tubes at the beginning and end of the study, were extracted, and the FA composition of the lipid fraction was analysed using GC.

Total lipids from 200 μl plasma (with 196 μg/ml tricosanoic acid (C23:0 FA, T653MG, Sigma-Aldrich) as internal standard) were extracted using acidic Folch extraction (in which 120 mM HCl in methanol was added prior to the addition of chloroform, to yield 1:2:4 v/v/v ratio of sample:methanol:chloroform, respectively)^(22)^. Samples were then sonicated in an ultra-sonication water bath for 5 min followed by phase separation by centrifugation at 2000 ***g*** for 5 min at 4°C. The organic phase was then collected and evaporated with N_2_ and further methylated for 2 h at 60°C using 1·25 M HCl in methanol (Sigma). The FA methyl esters were then further extracted using hexane. The organic phase was evaporated and redissolved in hexane for GC analysis. The separation and quantification of FA methyl esters were done using an SP-2560 column, 74 m × 0·18 mm (Supelco, Merck) in a GC-FID system (Agilent Technologies 7890B) programmed for a stepwise temperature elevation from 100 to 240°C. The peak areas were determined using Agilent ChemStation software, using the retention time and concentrations of known FAME standards (TraceCERT® Supelco 37 Component FAME Mix, CRM47885, Supelco, Sigma). Since the aim of the study was to investigate relative changes in the FA composition of total plasma lipids and not the quantitative levels of each FA of the plasma lipids (which would vary considerably with changes in individual total plasma lipids), the FA composition in mol% of total FA methyl esters was calculated. The SCD1 index (as a crude measurement of SCD1 activity) was estimated using the C16 : 1*n*-7:C16:0 ratio (because of the low abundance of C16 : 1*n*-7 in regular diets compared with C18 : 1*n*-9), and the D5D index (as a crude measurement of D5D activity) was estimated using the 20 : 4*n*-6:20 : 3*n*-6 ratio^(23)^.

### Clinical measurements for liver status, anthropometrics and plasma lipids

Magnetic resonance spectroscopy (using a 3T magnetic resonance scanner, Ingenia; Philips Healthcare) was carried out within 48 h before randomisation and at the end of the study. The magnetic resonance spectroscopy data were analysed using the Java-based magnetic resonance user interface software^(24)^ to obtain the percentage of fat in the liver. Liver stiffness was measured with FibroScan® (Echosens) transient elastography. For a detailed description of the magnetic resonance spectroscopy and elastography, see Holmer *et al.* ^(19)^. Fasting plasma total cholesterol (P-TC), plasma LDL-cholesterol, plasma HDL-cholesterol and plasma TAG (P-TAG) were analysed in the Clinical Chemistry Department of the Karolinska University Hospital laboratory. Anthropometric measures (weight and length) were measured at the beginning and at the end of the study. BMI was calculated (using the formula; weight(kg)/height squared(m^2^)).

### Statistics

The distributions of the variables (original values as mol% of plasma FA and E% of FA in the calculated food intake, fat % in liver, liver stiffness (kPa) and concentrations for total plasma lipids) were examined using the Shapiro–Wilk *W* test on the whole sample set. If *W* < 0·95, values were considered to be non-normally distributed and thus log transformed. For normally distributed values, a paired two-tailed Student’s *t* test was used for changes from baseline to end of the study. For non-normally distributed values after log transformation, the Wilcoxon matched-pair signed-rank test was used to detect changes after intervention in the different groups. For differences among the groups, one-way ANOVA (followed by Holm-Sidak’s multiple comparisons test) or Kruskal–Wallis (followed by Dunn’s multiple comparisons test) were used. Pearson’s correlation was used for normally distributed values. For non-normally distributed values (also after log transformation), Spearman was used for the correlation between changes in plasma FA composition and changes in reported FA intake. Changes in plasma FA composition and reported FA intake, respectively, were further used to investigate correlations between the differences in liver fat%, elastography, BMI and plasma lipids from the baseline to the end of the study. When a significant correlation between described parameters was found, multiple linear regression analysis was used to correct for changes in BMI during the study time. Changes in liver fat%, elastography and plasma lipids (P-TC, plasma LDL-cholesterol, plasma HDL-cholesterol and P-TAG) were used as dependent values, and changes in reported FA intake (as E%) or plasma FA composition (mol% of total) as independent variables and change in BMI were used as a covariate. A *P*-value of <0·05 was considered as significant for all statistical determinations. All data analysis was performed using GraphPad Prism 9.

## Results

### Changes in self-reported intake of fatty acids

Based on the data from the 3 d food records, the LCHF group significantly increased their relative energy (E%) intake of all FA, except for C12 : 0 and C20 : 5*n*-6. The 5:2 group reduced their E% intake of the majority of SFA, while no change was found for MUFA and PUFA. The SoC group did not change their FA E% intake, except for a decrease in reported intake of SCFA (C4:0–C10:0) (online Supplementary Table S1).

At the end of the study, the relative intake of cholesterol and SFA in the LCHF group was twice as high as in both the SoC and the 5:2 groups. The relative intake of MUFA was 37 % and 57 % higher in the LCHF group compared with SoC and 5:2, respectively, and the PUFA intake was 62 % higher in the LCHF group compared with the 5:2 group (Table 1).

**Table 1.** Fatty acid (FA) and cholesterol intake at the end of the intervention

Intake	SoC (*n* 15)		LCHF (*n* 15)		5:2 (*n* 18)		*P*
	Mean	95 % CI	Mean	95 % CI	Mean	95 % CI	
Total SFA	13·69	10·61, 18·55	28·17*	19·50, 37·28	11·32†	6·16, 16·05	<0·001
FA 4:0–10:0	0·91	0·33, 1·29	2·37*	0·78, 3·26	0·64†	0·16, 1·17	<0·001
FA 12:0	0·37	0·15, 0·87	0·91*	0·34, 3·72	0·26†	0·07, 0·74	<0·001
FA 14:0	1·34	0·68, 1·84	3·13*	1·61, 4·38	0·95†	0·24, 1·46	<0·001
FA 16:0	6·03	4·57, 8·87	13·98*	8·56, 16·74	5·67†	3·13, 8·48	<0·001
FA 18:0	2·45	1·73, 3·59	5·07*	3·15, 6·75	2·27†	0·99, 4·10	<0·001
FA 20:0	0·09	0·06, 0·14	0·12*	0·08, 0·23	0·08†	0·04, 0·19	0·012
Total MUFA	16·90	10·20, 22·17	23·12*	18·66, 32·85	14·73†	7·51, 23·08	<0·001
FA 16:1	0·77	0·37, 1·10	1·33*	0·65, 2·67	0·46	0·21, 1·30	<0·001
FA 18:1	12·77	8·10, 20·52	19·50*	14·50, 29·25	12·37	6·97, 21·89	<0·001
Total PUFA	6·31	4·53, 12·06	9·61	4·65, 16·77	5·87†	2·80, 9·10	0·050
FA 18:2	4·48	2·73, 7·77	5·72	3·25, 14·71	4·41	2·32, 7·27	0·054
FA 18:3	1·19	0·53, 3·39	1·02	0·64, 2·11	0·78	0·35, 1·94	0·084
FA 20:4	0·08	0·04, 0·21	0·13	0·07, 0·27	0·06†	0·02, 0·18	0·002
FA 20:5	0·13	0·00, 0·67	0·04	0·00, 0·64	0·02	0·00, 0·434	0·465
FA 22:5	0·05	0·01, 0·30	0·05	0·01, 0·29	0·02	0·00, 0·15	0·054
FA 22:6	0·18	0·01, 1·32	0·19	0·03, 1·23	0·11	0·02, 0·42	0·371
Cholesterol Density^1^	0·18	0·13, 0·23	0·34*	0·26, 0·42	0·17†	0·14, 0·20	<0·001

SoC = standard of care; LCHF = low-carbohydrate high fat.

FA intake (in E%) and ^1^cholesterol density (in g/1000 kcal) in the groups at the end of the study. Differences among groups were tested with Kruskal–Wallis.

* <0.05 for SoC *v*. LCHF.

† < 0.05 LCHF *v*. 5:2 for the *post hoc* test. No significant differences between SoC and 5:2 were seen.

### Changes in plasma fatty acid composition

From baseline to the end of the study, the LCHF group significantly increased the %mol of total *n*-6 PUFA (from 33·6 to 36·3 mol%), more specifically by increases in C18 : 2*n*-6 (from 26·2 to 28·3 mol%) and C20 : 4*n*-6 (from 6·0 to 7·4mol%). The LCHF group further decreased their relative plasma composition of total SFA, and C14 : 0 (from 35·2 to 33·7 and 0·9 to 0·4 mol% respectively) as well as the MUFA C16 : 1n-7 and the PUFA C20 : 3*n*-6 (from 2·4 to 1·7 % and 1·4 to 0·8 %, respectively) from baseline to end on the intervention. This contributed to a significant decrease in the SCD1 index (0·09–0·07) and an increase in the D5D index (4·7–6·8), respectively. The *n*-3:*n*-6 PUFA ratio decreased slightly (0·1–0·09) due to the relative increase in *n*-6 PUFA but unchanged levels of *n*-3 PUFA.

The 5:2 group also increased their relative content of plasma *n*-6 PUFA (32·6–34·2%); however, only C20 : 4*n*-6 was significantly increased (from 5·9 to 6·8%) among the individual FA of this group. Like the LCHF group, decreases in C16 : 1*n*-7 (2·5–2·1%), in C20 : 3*n*-6 (1·5–1·2 %) and in the SCD1 index (0·09–0·08) were seen, as well as an increase in the D5D index (3·8–5·0). For the SoC group, the plasma FA composition did not change from baseline to the end of the treatment (online Supplementary Table S3).

At the end of the study, the relative amount of total SFA and MUFA did not differ between the three groups. However, C16 : 1*n*-9 was lower in both the LCHF and 5:2 groups (1·71 (95 % CI 1·30–2·11) and 2·12 (95 % CI 1·72–2·53), respectively) compared with the SoC group (2·97 (95 % CI 2·50–3·43)). This further contributed to a lowered SCD1 index in both the LCHF and 5:2 group (0·07 (95 % CI 0·05–0·08)) and 0·08 (95 % CI 0·07–0·09), respectively) compared with the SoC group (0·11 (95 % CI 0·09–0·12)). Meanwhile, the relative amount of total PUFA did not differ between the three groups, C20 : 3*n*-6 was significantly higher in the 5:2 group (1·15 (95 % CI 0·87–1·43)) compared with the LCHF group (0·63 (95 % CI 0·34–0·92)) and C20 : 4*n*-6 was significantly higher in the LCHF group (7·35 (95 % CI 6·45–8·24)) compared with the SoC group (5·92 (95 % CI 5·10–6·74)), which further resulted in an increased D5D index for the LCHF group compared with the SoC group (Table 2). At baseline, no significant differences between the three groups were seen, with the exception of a lower SCD1 index in the 5:2 group compared with the LCHF group (online Supplementary Table S4).

**Table 2.** Fatty acid (FA) composition in plasma at the end of the intervention

FA in mol% of total plasma FA	SoC (*n* 15)		LCHF (*n* 15)		5:2 (*n* 18)		*P*
	Mean	95 % CI	Mean	95 % CI	Mean	95 % CI	
Sum of SFA	35·27	33·98, 36·56	33·70	32·67, 34·72	33·65	32·58, 34·72	0·063
C14 : 0	1·04	0·67, 1·42	0·49	0·31, 0·68	0·59	0·33, 0·85	0·057
C16 : 0	26·78	25·86, 27·71	26·04	25·11, 26·97	26·18	25·53, 26·83	0·378
C18 : 0	7·44	6·86, 8·03	7·16	6·56, 7·76	6·88	6·43, 7·34	0·296
Sum of MUFA	28·16	26·53, 29·79	26·28	24·01, 28·55	28·41	26·51, 30·31	0·217
C16 : 1*n*-7	2·97	2·50, 3·43	1·71*	1·30, 2·11	2·12†	1·72, 2·53	<0·001
C18 : 1*n*-9	25·19	23·70, 26·69	24·57	22·56, 26·59	26·29	24·44, 28·14	0·348
Sum of *n*-6 PUFA	32·86	30·44, 35·29	36·30	33·63, 38·98	34·18	32·34, 36·03	0·097
C18 : 2*n*-6	25·73	23·10, 28·36	28·30	25·78, 30·82	25·97	23·81, 28·13	0·232
C20 : 3*n*-6	1·09	0·73, 1·44	0·63	0·34, 0·92	1·15‡	0·87, 1·43	0·013
C20 : 4*n*-6	5·92	5·10, 6·74	7·35*	6·45, 8·24	6·99	6·14, 7·85	0·049
Sum of *n*-3 PUFA	3·74	2·74, 4·62	3·02	1·80, 7·37	3·68	1·89, 5·91	0·446
C18 : 3*n*-3	0·59	0·39, 0,79	0·51	0·23, 0·78	0·49	0·32, 0·66	0·689
C20 : 5*n*-3	1·02	0·79, 1·26	0·51	0·20, 2·68	0·79	0·48, 1·09	0·078
C22 : 6*n*-3	2·08	1·91, 2·24	2·32	1·81, 2·83	2·46	2·00, 2·91	0·498
Sum of LA and ALA	26·32	23·63, 29·00	28·81	26·23, 31·39	26·46	24·28, 28·64	0·202
**Index (ratios)**							
*n*-3:*n*-6	0·12	0·08, 0·16	0·09	0·05, 0·21	0·10	0·05, 0·18	0·254
SCD1 (16 : 1:16 : 0)	0·11	0·09, 0·12	0·07*	0·05, 0·08	0·08 (0·07, 0·09)†		<0·001
D5D (20 : 4:20 : 3)	4·42	3·16, 5·68	7·72*	5·57, 9·87	5·76	4·56, 6·96	0·005

SoC = standard of care; LCHF = low-carbohydrate high fat; ALA = α-Linolenic acid (C18:3*n*-3); LA = Linoleic acid (C18:2*n*-6); SCD1 = stearoyl-CoA desaturase; D5D = δ5-desaturase.

Normally distributed values were tested for differences between groups using one-way ANOVA, and for non-normally distributed values, Kruskal–Wallis was used.

* <0.05 for SoC *v*. LCHF.

† <0.05 for SoC *v*. 5:2.

‡ < 0.05 LCHF *v*. 5:2 for the *post hoc* test.

### Correlation between reported fatty acid intake and plasma fatty acid composition

**Fig. 1.**
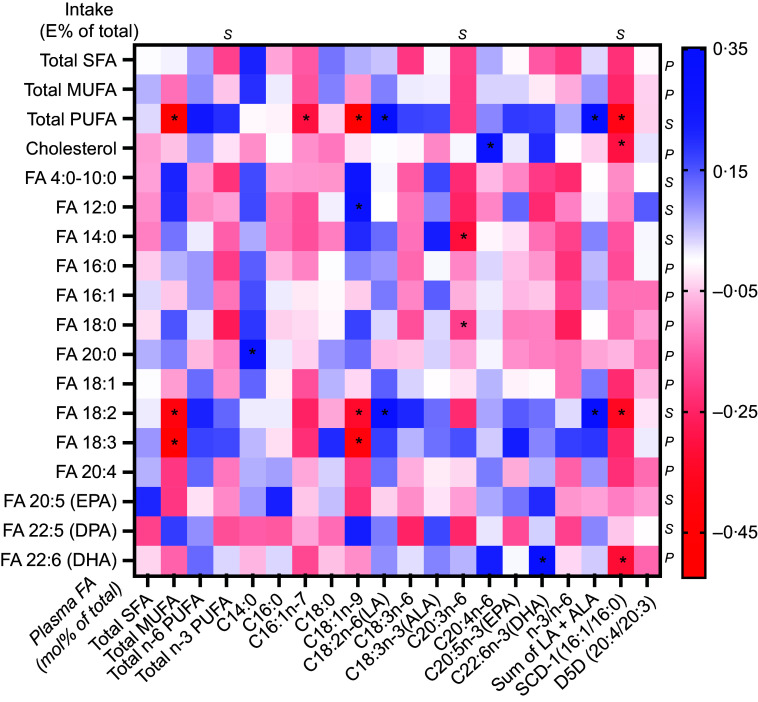
Correlations between change in intake of fatty acids (FA) (in E% of total E) and cholesterol density (g/1000 kcal) with plasma FA (mol% of total FA) or the index of substrate to FA products corresponding to enzyme activities for stearoyl-CoA desaturase 1 (SCD1) and δ5-desaturase (D5D). For normally distributed values (before or generated after log transformation), Pearson’s correlation (*P*) was used; for values that still did not reach normal distribution after log transformation, Spearman’s correlations (*S*) were used. * = Significant correlation *P* < 0·05.

Changes in the reported intake of total PUFA and intake of C18 : 2*n*-6 correlated positively with changes in plasma C18 : 2*n*-6 (*rho* 0·34; *P* = 0·016 and 0·31; *P* = 0·031, respectively) and the sum of plasma C18 : 2*n*-6 and C18 : 3*n*-3 (*rho* 0·35; *P* = 0·013 and 0·31; *P* = 0·027) (Fig. 1). Changes in the intake of total PUFA and C18 : 2*n*-6 alone inversely correlated with changes in total plasma MUFA (*rho* −0·52; *P* < 0·001 and −0·40; *P* = 0·004), C18 : 1*n*-9 (*rho* −0·44; *P* = 0·001 and −0·33; *P* = 0·020) and the SCD1 index (*rho* −0·38; *P* < 0·007 and −0·35; *P* = 0·014). The reported change in the intake of the *n*-3 PUFA C22 : 6*n*-3 was positively correlated with the change of plasma C22 : 6*n*-3 (*r* 0·32; *P* = 0·027) and was inversely correlated with the change in plasma SCD1 index (–0·32; *P* = 0·027).

The change in reported cholesterol intake demonstrated a positive correlation with changes in plasma C20 : 4*n*-6 (*r* 0·29; *P* = 0·045) and an inverse correlation with changes in the SCD1 index (*rho* −0·30; *P* = 0·039) (Fig. 1). Changes in the reported intake of total SFA or MUFA did not show any correlation with changes in specific plasma FA species, total plasma FA in respective FA group or calculated indices.

### Association of changes in reported fatty acid intake to changes in liver and plasma parameters

**Fig. 2.**
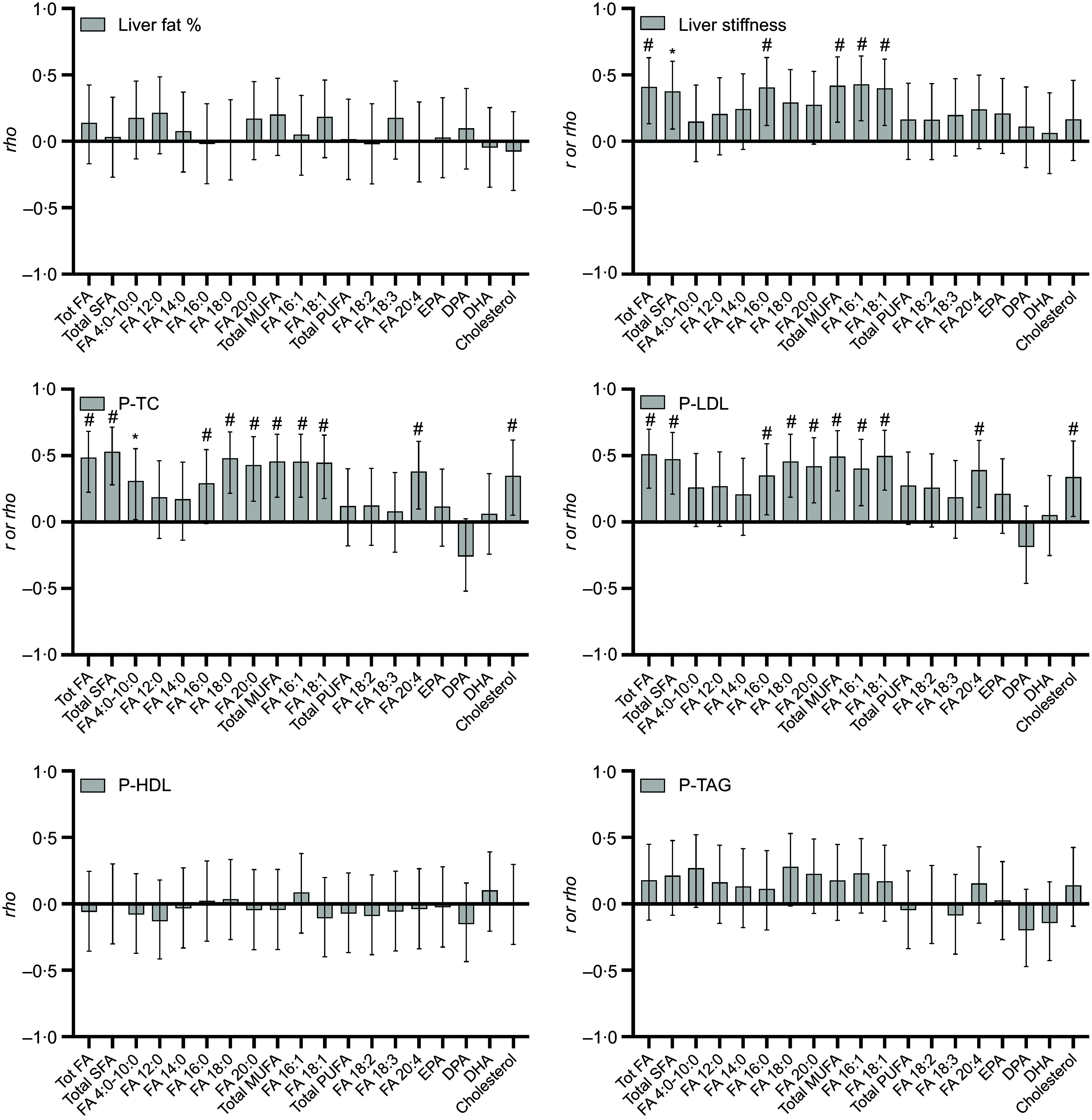
Correlations between change in reported fatty acid (FA) and cholesterol intake (in E% of total E and cholesterol density g/1000g) and change in liver fat, liver stiffness and different plasma total cholesterol (P-TC), plasma LDL-cholesterol (P-LDL), plasma HDL-cholesterol (P-HDL) and TAG (P-TAG) in forty-five participants (SoC *n* 14, LCHF *n* 13, 5:2 *n* 18). The bars represent Pearson’s *r* or Spearman’s *rho* values (with 95 % CI). * = significant correlation *P* < 0·05, # = significant correlation *P* < 0·01.

Change in liver fat percentage was not associated with any changes in self-reported intake of total, specific or groups of FA. However, change in liver stiffness was positively associated with changes in reported intake of total FA (*r* 0·41; *P* = 0·005), total SFA (*r* 0·38; *P* = 0·011), total MUFA (*r* 0·42; *P* = 0·004), C16 : 0 (*rho* 0·41; *P* = 0·003), C16 : 1 (*r* 0·43; *P* = 0·005) and C18 : 1 (*r* 0·40; *P* = 0·007). These associations remained significant when adjusting for change in BMI (Fig. 2 and Table 3).

**Table 3.** Associations between changes in reported fatty acid (FA) intake and changes of elastography and plasma lipids

Dependent parameter	Variable FA (E%)	Multilinear reg. (+BMI (kg/m^2^)) estimated *β*	95 % CI	*P*
Elastography (kPa)	Total FA	0·27	0·07, 0·47	0·009
	Total SFA	0·20	0·04, 0·35	0·016
	Total MUFA	0·27	0·08, 0·46	0·007
	FA 16:0	0·20	0·05, 0·36	0·012
	FA 16:1	0·18	0·06, 0·30	0·005
	FA 18:1	0·24	0·06, 0·42	0·011
				
Plasma total cholesterol (mmol/l)	Total FA	1·58	0·76, 2·40	<0·001
	Total SFA	1·29	0·67, 1·92	<0·001
	Total MUFA	1·45	0·64, 2·26	0·001
	FA 4:0–10:0	0·41	0·02, 0·79	0·040
	FA 16:0	1·19	0·54, 1·83	0·001
	FA 18:0	1·14	0·52, 1·75	0·001
	FA 20:0	1·10	0·46, 1·75	0·001
	FA 16:1	0·89	0·37, 1·41	0·001
	FA 18:1	1·38	0·62, 2·15	0·001
	FA 20:4	0·73	0·21, 1·25	0·008
	Total cholesterol	0·88	0·22, 1·53	0·010
				
Plasma LDL-cholesterol (mmol/l)	Total FA	0·21	0·10, 0·32	<0·001
	Total SFA	0·15	0·06, 0·23	0·001
	Total MUFA	0·19	0·09, 0·30	0·001
	FA 16:0	0·15	0·06, 0·24	0·001
	FA 18:0	0·14	0·05, 0·22	0·002
	FA 20:0	0·13	0·04, 0·22	0·004
	FA 16:1	0·10	0·03, 0·17	0·008
	FA 18:1	0·19	0·09, 0·29	0·001
	FA 20:4	0·09	0·02, 0·16	0·010
	Total cholesterol	0·12	0·03, 0·21	0·008

Multiple linear regression of absolute change in elastography and plasma lipids with changes in intake of different FA intake (E%) and cholesterol density (g/1000 kcal), with change in BMI as a covariate. *n* 45 (SoC *n* 14, LCHF *n* 13, 5:2 *n* 18). Only significant associations in the correlation analysis were analysed. SoC = standard of care; LCHF = low-carbohydrate high fat.

Change in P-TC was positively correlated with changes of reported intake of cholesterol (*rho* 0·35; *P* = 0·020), total amount of FA (*r* 0·39; *P* < 0·001), SFA (*r* 0·53; *P* < 0·001), MUFA (*r* 0·46; *P* = 0·002) and most of the reported specific FA for these two groups. The only PUFA with a significant association with a change in plasma cholesterol was C20 : 4*n*-6 (*r* 0·38; *P* = 0·010) (Fig. 2). The change in LDL-cholesterol was positively associated with the intake of cholesterol (*rho* 0·34; *P* = 0·023), total intake of FA (*r* 0·51; *P* < 0·001), total SFA (*r* 0·47; *P* = 0·001) and the specific SFA C16 : 0–C20 : 0, total MUFA (*r* 0·49; *P* < 0·001) and the specific MUFA C16 : 1 and C18 : 1 (Fig. 2). All these associations remained significant after adjusting for change in BMI (Table 3). No significant associations between reported changes in FA intake and plasma HDL-cholesterol or changes in P-TAG were seen.

### Association of changes in plasma fatty acid composition to changes in liver and plasma parameters

**Fig. 3.**
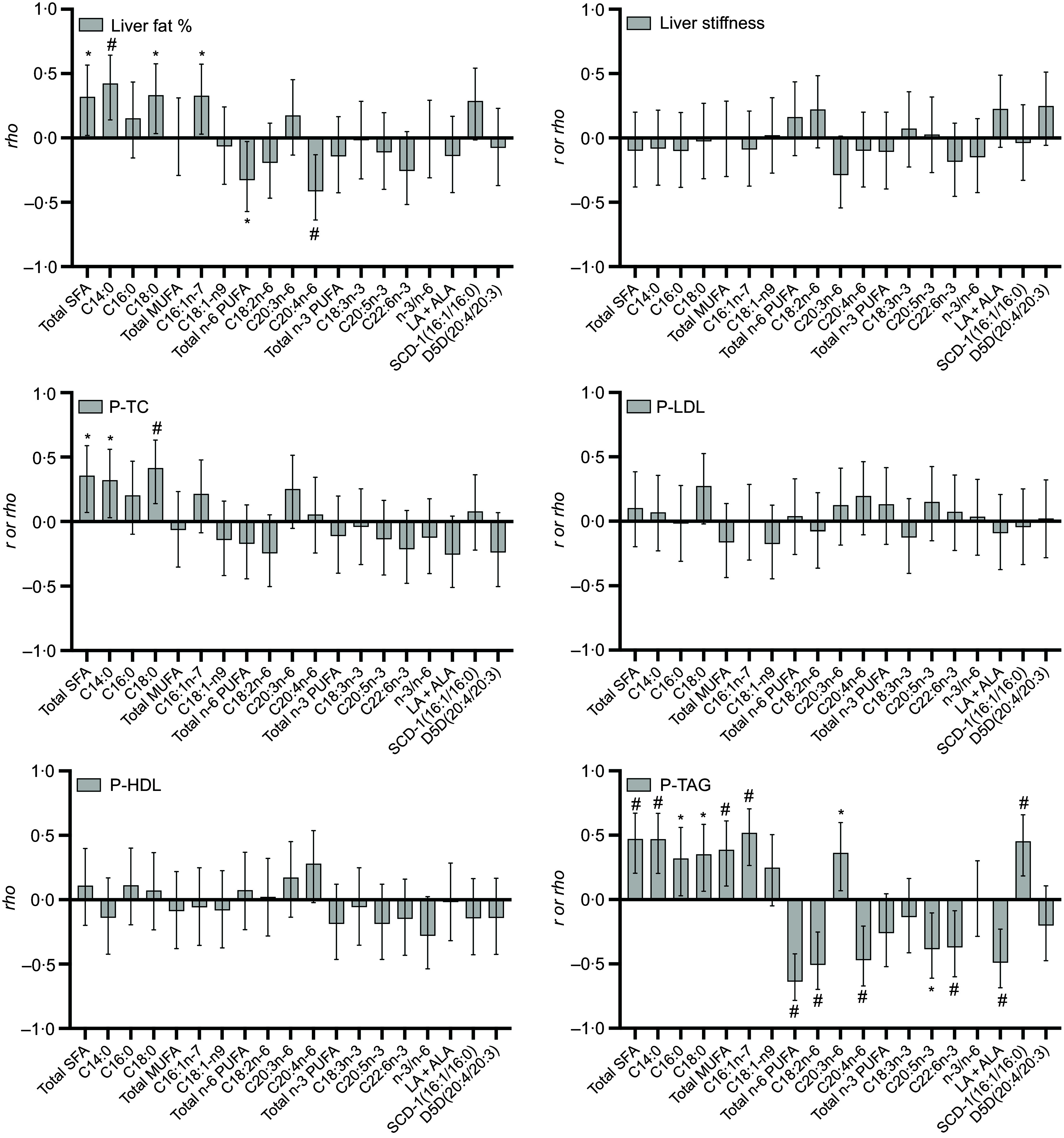
Correlations between change in fatty acid (FA) composition (mol% of total plasma FA), stearoyl-CoA desaturase, δ5-desaturase (D5D) and *n*3/*n*6 indices and change in liver fat, liver stiffness, plasma total cholesterol (P-TC), plasma LDL-cholesterol (P-LDL), plasma HDL-cholesterol (P-HDL) and TAG (P-TAG), respectively) in forty-five participants (SoC *n* 14, LCHF *n* 13, 5:2 *n* 18). The bars represent Pearson’s *r* and Spearman’s *rho* values (with 95 % CI).* = significant correlation *P* < 0·05, # = significant correlation *P* < 0·01.

Changes in liver fat percentage from baseline to the end of the study were positively associated with changes in the proportion of plasma total SFA (*r* 0·32; *P* = 0·032), C18 : 0 (*r* 0·33; *P* = 0·026) and C14 : 0 (*r* 0·42; *P* = 0·004) and with increased plasma proportions of 16 : 1*n*-7 (*r* 0·33; *P* = 0·028). However, although a positive trend was noted (*P* = 0·055), the SCD1 index did not reach significance. Meanwhile, an inverse correlation between changes in liver fat and changed proportions of plasma total *n*-6 PUFA (*r* –0·33; *P* = 0·028) and C20 : 4*n*-6 (*r* −0·42; *P* = 0·005) was seen (Fig. 3). Except for C18 : 0, significant associations in the correlation analysis remained significant after adjusting for change in BMI (Table 4).

**Table 4.** Associations between change in plasma fatty acid (FA) composition with changes in liver fat and plasma lipids

Dependent parameter	Variable *P*-FA (mole %)	Multilinear reg. (+BMI (kg/m^2^)) estimated *β*	95 % CI	*P*
Liver fat%	SFA	0·04	0·01, 0·06	0·006
	*n*-6 PUFA	–0·04	–0·06, −0·01	0·006
	C14 : 0	0·36	0·14, 0·57	0·002
	C18 : 0	0·07	–0·01, 0·14	0·090
	C16 : 1*n*-7	0·19	0·09, 0·30	<0·001
	C20 : 4*n*-6	–1·34	–2·17, −0·49	0·003
				
Plasma TC (mmol/l)	SFA	0·08	0·01, 0·15	0·018
	C14 : 0	0·64	0·07, 1·21	0·029
	C18 : 0	0·26	0·08, 0·44	0·005
				
Plasma TAG (mmol/l)	SFA	0·02	0·01, 0·04	0·001
	MUFA	0·02	0·01, 0·04	0·010
	*n*-6 PUFA	–0·03	–0·04, −0·02	<0·001
	Sum LA + ALA	–0·02	–0·04, −0·01	<0·001
	C14 : 0	0·20	0·08, 0·31	0·001
	C16 : 0	0·02	0·00, 0·05	0·033
	C16 : 1*n*-7	0·11	0·05, 0·16	<0·001
	C18 : 0	0·05	0·01, 0·09	0·019
	C18 : 2*n*-6	–0·03	–0·04, −0·01	<0·001
	C20 : 3*n*-6	0·32	0·05, 0·59	0·021
	C20 : 4*n*-6	–0·79	–1·22, −0·35	0·001
	C20 : 5*n*-3	–0·21	–0·37, −0·05	0·010
	C22 : 6*n*-3	–0·38	–0·67, −0·09	0·012
	SCD1 (16 : 1:16 : 0)	0·51	0·20, 0·82	0·002

TC = total cholesterol; SCD1 = stearoyl-CoA desaturase 1; SoC = standard of care; LCHF = low-carbohydrate high fat.

Multiple linear regression of absolute change in liver fat and plasma lipids with changes in different plasma FA (mole %), with BMI as a covariate. *n* 45 (SoC *n* 14, LCHF *n* 13, 5:2 *n* 18). Only significant associations in the correlation analysis were analysed.

No significant associations between changes in liver stiffness and changes in plasma FA composition were seen (Fig. 3).

Change in the total amount of SFA (*r* 0·36; *P* = 0·016), C14 : 0 (*r* 0·33; *P* = 0·032) and C18 : 0 (*r* 0·42; *P* = 0·005) correlated positively with changes in total plasma cholesterol, and this association remained significant in the regression model. However, neither change in LDL-cholesterol nor HDL-cholesterol showed any correlation with changes in plasma FA (Table 4). Change in total proportions of plasma SFA (*r* 0·47; *P* = 0·001) (and C14 : 0 (*r* 0·47; *P* = 0·001) and C18 : 0 (*r* 0·35; *P* = 0·012)) and total MUFA (*r* 0·39; *P* = 0·009) (and C16 : 1*n*-7 (*r* 0·51; *P* < 0·001) specifically), as well as the SCD1 index (*r* 0·45; *P* = 0·002), was positively correlated with change in P-TAG. An inverse correlation with the change in P-TAG and increased proportions of C22 : 6*n*-3 (*r* −0·37; *P* = 0·012) and C20 : 5*n*-3(*r* −0·39; *P* = 0·009) and of total *n*-6 PUFA (*r* −0·64; *P* < 0·001), C18 : 2*n*-6 (*r* −0·51; *P* < 0·001) and C20 : 4*n*-6 (*r* –0·47; *P* = 0·005)) was found. All mentioned associations stayed significant after adjusting for change in BMI (Fig. 3, Table 4).

## Discussion

In this secondary analysis of a randomised controlled trial, we found that an increased dietary intake of PUFA increased plasma PUFA and that increasement of plasma *n*-6 PUFA was associated with larger liver fat loss. In addition, both increases in dietary intake SFA and increased content of plasma SFA were associated with less reduction in liver stiffness and liver fat loss, respectively.

Correlation analysis between dietary intake and plasma composition of FA showed that increased intake of PUFA correlated positively with plasma PUFA and inversely to plasma MUFA. Intake of PUFA is known to correlate with the plasma content of PUFA and in particular with the two essential FA: C18 : 2*n*-6 (linoleic acid) and C18 : 3*n*-3 (α-linolenic acid)^(25–29)^. The LCHF group increased their plasma content of C18 : 2*n*-6^(19,20)^. This is in accordance with an earlier observation that a diet high in PUFA, but also high in total fat, increases C18 : 2*n*-6 in plasma lipids^(27–30)^.

Although there was a large difference in fat intake between the groups, where LCHF had significantly higher dietary FA intake in particular of SFA and MUFA, only a few significant changes in plasma FA composition were seen between the groups after 12 weeks. When analysing the whole study population, increased reported intake of total fat, SFA and MUFA were associated with a lower reduction in liver stiffness during this study. An intake of a high amount of total fat and SFA (and diets poor in PUFA) has previously been associated with increased intrahepatic lipid accumulation and with more severe stages of NAFLD^(31,32)^. In particular, increased intake of C16 : 0, C18 : 0 and C16 : 1 were shown to correlate with increased liver stiffness in NAFLD patients. High intake of C18 : 2*n*-6 has also been shown to be associated with NAFLD severity^(18)^. In our study, neither intake of C18 : 2*n*-6 nor any other PUFA were associated with any change in liver stiffness. However, in the mentioned study, the patients with more advanced stages of NAFLD also tended to report higher energy and total fat (g/d) intake, which may have contributed to the association between C18 : 2*n*-6 and a more progressive state of NAFLD^(18)^. Cellular accumulation of SFA is known to be lipotoxic – that is, triggering endoplasmic stress and inducing pro-inflammatory pathways^(33,34)^ – effects that promote the progression of NAFLD to more severe states. Furthermore, SFA are substrates for bioactive lipid intermediates such as diacylglycerols and ceramides that contribute to hepatic insulin resistance^(35)^. Hyperenergetic intervention studies with high intake of SFA have been shown to increase both circulating and hepatic ceramides^(29,36)^. Increased intake of MUFA and its connection to NAFLD development, or progression of the disease, are less clear in the literature. However, increased intake of total fat usually also correlates with a high intake of MUFA^(37)^. It is hypothesised that the desaturation of SFA into MUFA would promote the channelling of these FA into phospholipids and TAG, thereby preventing the accumulation of lipotoxic SFA. In a recent cross-sectional study investigating the FA content of different hepatic and plasma lipid species, biopsy-proven fibrosis was associated with a lower amount of MUFA containing TAG in both the liver and plasma; however, data regarding intake of FA was not evaluated herein^(38)^.

Interestingly, changes in reported intake of total FA, groups or individual FA were not associated with changes in liver fat, but changes in plasma FA composition were associated with changes in liver fat. An increase in plasma *n*-6 PUFA, especially C20 : 4*n*-6, was associated with a larger loss of liver fat, whereas SFA, especially FA C14:0 and C18:0, were associated with a lower loss of liver fat. A high intake and high plasma lipid content of *n*-6 PUFA have been associated with a lower amount of liver fat^(14–17,39,40)^. Furthermore, diets rich in PUFA, compared with SFA-rich diets, have been shown to promote liver fat loss in hypoenergetic intervention studies^(26,41)^ and to be protective against hepatic fat accumulation during hyperenergetic intervention studies^(28,29,36)^. However, not all such studies have seen similar effects^(42)^. Most of the mentioned studies showing the beneficial effects of *n*-6 PUFA point out C18 : 2*n*-6 as a main driver of the protective effect of *n*-6 PUFA in NAFLD. Contradictive to what was seen in this study, the plasma *n*-6 PUFA C20 : 4*n*-6 have been shown to be associated with an increased amount of liver fat^(14)^. However, common findings in studies where the hepatic lipidome has been investigated in subjects with steatosis or different stages of NAFLD are increased alterations in the hepatic content of long-chain PUFA^(43–47)^. In addition, the D5D index, a crude measurement of the endogenous conversion of 20 : 3*n*-6 to C20 : 4*n*-6, was significantly increased in the LCHF group (and non-significantly increased in the 5:2 group) compared with the SoC group. It is therefore tempting to hypothesise that the association of increased plasma C20 : 4*n*-6 with increased loss of hepatic fat found in this study could be a sign of improved endogenous synthesis of C20 : 4*n*-6, especially since no correlation between change in intake of C20 : 4 and change in plasma C20 : 4*n*-6 was seen in this study. Further studies need to be conducted to evaluate if our finding of increased plasma C20 : 4*n*-6 could be a consequence of the reestablishment of hepatic PUFA synthesis after a reduction in liver lipid accumulation has occurred.

Some potential protective effects of *n*-3 PUFA or an elevated *n*-3 PUFA to *n*-6 PUFA ratio in the pathogenesis of NAFLD have been reported^(48)^. Despite a positive correlation observed between the altered intake of C22 : 6*n*-3 and the modified proportions of plasma C22 : 6*n*-3, no additional associations were identified between the intake or plasma levels of C22 : 6*n*-3, or any other investigated *n*-3 PUFA, and changes in liver fat or liver stiffness in this study.

A higher rate of hepatic DNL has been reported in patients with NAFLD and obesity^(8)^ and increased mRNA expression of hepatic SCD1 has been found^(40,49)^. Although no significant association between the change in the SCD1 index and change in liver fat was seen, an increase in C16 : 1*n*-7, a main product for SCD1 activity, was associated with a lower reduction of liver fat in this study. C16 : 1*n*-7 only exists in low amounts in regular foods, and the majority of C16 : 1*n*-7 found in plasma is produced by DNL in the liver^(36,50,51)^. Both intervention groups had a larger reduction of liver fat and a reduced SCD1 index compared with the SoC group. Further in line with our findings, increased plasma C16 : 1 and/or SCD1 index have been associated with an increased amount of liver fat in both healthy individuals and patients diagnosed with NAFLD^(8,14,17,40,52)^. An increased SCD1 index in blood lipids has also been positively associated with liver fat content in controlled hyperenergetic^(28,29)^, isoenergetic^(53)^ and hypoenergetic intervention studies^(26,41)^.

In the present study, changes in intake of SFA, MUFA, C20 : 4*n*-6 and cholesterol associated positively with changes in P-TC and LDL-cholesterol. However, only changes in plasma SFA (and C14 : 0 and C18 : 0 alone) were associated significantly with P-TC. Partial replacement of SFA to MUFA and preferably also with PUFA in a normal Western-type diet is known to reduce P-TC and LDL-cholesterol^(54,55)^. Although a high intake of SFA is paramount in promoting high P-TC and LDL-cholesterol, excess intake of cholesterol has also been associated with increased P-TC and LDL-cholesterol^(6,55–57)^. The main dietary sources of both cholesterol and C20 : 4*n*-6 are of animal origin such as eggs and meat^(58)^, which possibly explain the positive association of calculated C20 : 4*n*-6 intake to plasma cholesterol content seen in this study.

Positive associations of plasma SFA C16 : 1*n*-7 and the SCD1 index with P-TAG were seen in the present study. The plasma SCD1 index and DNL index have previously been associated with an increased P-TAG^(59–61)^. A change in intake of C18 : 2*n*-6 and C22 : 6*n*-3 correlated with a change of respective plasma FA. Although no association between an intake of different PUFA and changes in P-TAG was observed, a negative association of increased plasma C18 : 2*n*-6, C20 : 4*n*-6, C20 : 5*n*-3 and C22 : 6*n*-3 and reduction of plasma total TAG were found in this study. An increased amount of PUFA, both *n*-6 PUFA and especially *n*-3 PUFA, are known to reduce P-TAG via different mechanisms, that is, by reducing DNL, increasing FA oxidation and increasing extrahepatic VLDL catabolism^(62–64)^.

The strengths of this study are that the study participants represent a general NAFLD population and that it is one of the few studies that measures remission of liver fat in relation to both different E% intake of fats and changes in plasma FA composition. This study was not primarily designed to investigate the associations between dietary intake and plasma FA composition, and the main aim of the FA composition analysis was to get a crude measurement of the adherence to the diets (by investigating changes in C18 : 2*n*-6 and C18 : 3*n*-3 in the groups). This contributed to the main limitation of this study, that is, the low number of individuals in the three groups. The duration of the study was relatively short, 12 weeks, so no conclusions regarding the long-term effects of these changes can be drawn. To determine FA intake, self-reported dietary intake was used, and although this has limitations in its reliability, the total fat intake reported by the intervention groups was as intended for the groups^(20)^. In addition, increases in the total relative amount of C18 : 2*n*-6 and C18 : 3*n*-3 were seen in the LCHF group, as expected, confirming that the participants were adherent to the diet.

### Conclusions

This study shows that, in addition to energy restriction and weight loss, choices of dietary FA intake may be of importance for persons with NAFLD. When analysing the whole study population, in which dietary fat content differed considerably between study groups, we found that reducing the intake of total fat and especially SFA could have beneficial effects on liver stiffness and plasma cholesterol. Furthermore, an increased intake of both *n*-3 and *n*-6 PUFA affects the plasma composition of FA, which also may be beneficial for the reduction of liver fat, plasma cholesterol levels and TAG.

## Supporting information

Tillander et al. supplementary material Tillander et al. supplementary material

## Supplementary material

For supplementary material/s referred to in this article, please visit https://doi.org/10.1017/S0007114524001673.
