# Supplementary material for: Associations between dietary fatty acid and plasma fatty acid composition in non-alcoholic fatty liver disease: secondary analysis from a randomised trial with a hypoenergetic low-carbohydrate high-fat and intermittent fasting diet
Source: Br J Nutr. 2024 Sep 18;132(4):453–65. doi: 10.1017/S0007114524001673 (PMC11499086; doi:10.1017/S0007114524001673)
Supplement: Tillander et al. supplementary material [file S0007114524001673sup001.docx]

**Associations between dietary fatty acid and plasma fatty acid composition in non-alcoholic fatty acid liver disease – secondary analysis from a randomised trial with a hypocaloric low-carbohydrate high-fat and intermittent fasting diet**

***Supplementary material.***

**Table I.** Fatty acids and cholesterol energy percent at baseline and end of the study

|  | **Baseline** | **End** |  |
| --- | --- | --- | --- |
| **SoC (n=15)** | Median(95% CI) | Median(95% CI) | *p-val* |
| Total SFA | 15.83 (11.92-21.77) | 13.69 (10.61-18.55) | 0.083 |
| FA 4:0-10:0 | 1.11 (0.30-1.97) | 0.91 (0.33-1.29) | 0.035 |
| FA 12:0 | 0.57 (0.14-1.29) | 0.37 (0.15-0.87) | 0.055 |
| FA 14:0 | 1.55 (0.82-2.26) | 1.34 (0.68-1.84) | 0.055 |
| FA 16:0 | 7.27 (5.22-10.02) | 6.03 (4.57-8.87) | 0.083 |
| FA 18:0 | 2.83 (1.86-4.49) | 2.45 (1.73-3.59) | 0.151 |
| FA 20:0 | 0.10 (0.068-0.13) | 0.09 (0.06-0.14) | 0.524 |
| Total MUFA | 14.10 (10.09-21.75) | 16.90 (10.20-22.17) | 0.524 |
| FA 16:1 | 0.61 (0.39-0.98) | 0.77 (0.37-1.10) | 0.303 |
| FA 18:1 | 13.01 (8.51-18.56) | 12.77 (8.10-20.52) | 0.804 |
| Total PUFA | 4.93 (3.25-9.24) | 6.31 (4.53-12.06) | 0.208 |
| FA 18:2 | 3.78 (2.33-6.58) | 4.48 (2.73-7.77) | 0.330 |
| FA 18:3 | 0.94 (0.47-1.66) | 1.19 (0.53-3.39) | 0.095 |
| FA 20:4 | 0.07 (0.03-0.17) | 0.08 (0.04-0.21) | 0.679 |
| FA 20:5 | 0.03 (0.00-0.42) | 0.13 (0.00-0.67) | 0.330 |
| FA 22:5 | 0.02 (0.00-0.18) | 0.05 (0.01-0.30) | 0.639 |
| FA 22:6 | 0.08 (0.03-0.75) | 0.18 (0.01-1.32) | 0.561 |
| Cholesterol | 0.17 (0.14-0.21) | 0.17 (0.11-0.27) | 0.890 |
| **LCHF (n=15)** | Median(95% CI) | Median(95% CI) | *p-val* |
| Total SFA | 15.16 (6.71-22.90) | 28.17 (19.50-37.28) | **<0.001** |
| FA 4:0-10:0 | 1.03 (0.19-2.11) | 2.37 (0.78-3.26) | **0.002** |
| FA 12:0 | 0.56 (0.11-1.98) | 0.91 (0.34-3.72) | 0.107 |
| FA 14:0 | 1.33 (0.38-2.52) | 3.13 (1.61-4.38) | **0.001** |
| FA 16:0 | 6.85 (3.15-10.24) | 13.98 (8.56-16.74) | **<0.001** |
| FA 18:0 | 2.57 (1.12-4.46) | 5.07 (3.15-6.75) | **0.001** |
| FA 20:0 | 0.08 (0.03-0.12) | 0.12 (0.08-0.23) | **0.003** |
| Total MUFA | 13.21 (7.70-16.51) | 23.12 (18.66-32.85) | **<0.001** |
| FA 16:1 | 0.54 (0.18-0.92) | 1.33 (0.65-2.67) | **<0.001** |
| FA 18:1 | 11.29 (6.44-15.34) | 19.50 (14.50-29.25) | **<0.001** |
| Total PUFA | 4.52 (2.90-6.11) | 9.61 (4.65-16.77) | **<0.001** |
| FA 18:2 | 3.14 (2.09-4.63) | 5.72 (3.25-14.71) | **<0.001** |
| FA 18:3 | 0.71 (0.32-1.05) | 1.02 (0.64-2.11) | **0.007** |
| FA 20:4 | 0.05 (0.03-0.19) | 0.13 (0.07-0.27) | **<0.001** |
| FA 20:5 | 0.01 (0.00-0.45) | 0.04 (0.00-0.64) | 0.083 |
| FA 22:5 | 0.01 (0.00-0.20) | 0.05 (0.01-0.29) | **0.001** |
| FA 22:6 | 0.04 (0.00-1.03) | 0.19 (0.03-1.23) | **0.026** |
| Cholesterol | 0.18 (0.09-0.24) | 0.32 (0.24-0.46) | **0.003** |
| **5:2 (n=18)** | Median(95% CI) | Median(95% CI) | *p-val* |
| Total SFA | 15.73 (11.57-19.40) | 11.32 (6.16-16.05) | **0.001** |
| FA 4:0-10:0 | 1.03 (0.42-1.86) | 0.64 (0.16-1.173) | **0.003** |
| FA 12:0 | 0.56 (0.17-1.70) | 0.26 (0.07-0.738) | **0.001** |
| FA 14:0 | 1.36 (0.63-2.46) | 0.95 (0.24-1.459) | **0.001** |
| FA 16:0 | 7.34 (3.86-9.50) | 5.67 (3.13-8.479) | **0.012** |
| FA 18:0 | 2.63 (1.78-3.93) | 2.27 (0.99-4.098) | **0.034** |
| FA 20:0 | 0.11 (0.07-0.17) | 0.08 (0.04-0.190) | 0.246 |
| Total MUFA | 15.27 (11.81-21.06) | 14.73 (7.51-23.08) | 0.393 |
| FA 16:1 | 0.58 (0.22-1.03) | 0.46 (0.21-1.302) | 0.523 |
| FA 18:1 | 14.31 (10.38-18.48) | 12.37 (6.97-21.89) | 0.442 |
| Total PUFA | 5.32 (4.33-7.68) | 5.87 (2.80-9.10) | 0.495 |
| FA 18:2 | 3.99 (2.99-6.25) | 4.41 (2.32-7.27) | 0.304 |
| FA 18:3 | 0.96 (0.50-1.51) | 0.78 (0.35-1.94) | 0.702 |
| FA 20:4 | 0.07 (0.02-0.12) | 0.06 (0.02-0.18) | 0.702 |
| FA 20:5 | 0.03 (0.00-0.28) | 0.02 (0.00-0.43) | 0.678 |
| FA 22:5 | 0.01 (0.00-0.19) | 0.02 (0.00-0.15) | 0.832 |
| FA 22:6 | 0.11 (0.02-0.61) | 0.11 (0.02-0.42) | 0.640 |
| Cholesterol | 0.15 (0.11-0.19) | 0.16 (0.12-0.22) | 0.551 |

*Fatty acid energy % of total E intake for FAs and cholesterol density (g/1000kcal). Values presented as median, and 95% confidence intervals. Changes between baseline and end were tested using Wilcoxon paired test.*

**Table II.** Fatty acids and cholesterol energy percent intake at baseline

|  | **SoC (n=15)** | **LCHF (n=15)** | **5:2 (n=18)** | ***p-val*** |
| --- | --- | --- | --- | --- |
| Total SFA | 15.84 | 15.17 | 15.73 | 0.772 |
| FA 4:0-10:0 | 1.11 | 1.03 | 1.03 | 0.599 |
| FA 12:0 | 0.57 | 0.56 | 0.56 | 0.768 |
| FA 14:0 | 1.55 | 1.33 | 1.36 | 0.392 |
| FA 16:0 | 7.27 | 6.85 | 7.34 | 0.442 |
| FA 18:0 | 2.83 | 2.57 | 2.63 | 0.430 |
| FA 20:0 | 0.10 | 0.08 | 0.11^#^ | **0.048** |
| Total MUFA | 14.10 | 13.22 | 15.28 | 0.054 |
| FA 16:1 | 0.61 | 0.54 | 0.58 | 0.785 |
| FA 18:1 | 13.02 | 11.29 | 14.32^#^ | **0.024** |
| Total PUFA | 4.93 | 4.52 | 5.32 | 0.208 |
| FA 18:2 | 3.78 | 3.14 | 3.99 | 0.121 |
| FA 18:3 | 0.94 | 0.71 | 0.96 | 0.070 |
| FA 20:4 | 0.07 | 0.05 | 0.07 | 0.501 |
| FA 20:5 | 0.03 | 0.01 | 0.03 | 0.698 |
| FA 22:5 | 0.02 | 0.01 | 0.01 | 0.184 |
| FA 22:6 | 0.08 | 0.04 | 0.11 | 0.298 |
| Cholesterol | 0.19 | 0.24 | 0.14 | 0.539 |

*Differences in FA-composition of E% between groups at the end of the study were tested with Kruskal Wallice. # < 0.05 LCHF vs 5:2 for the post-hoc test. No other differences between the groups were seen with the post-hoc test.*

**Table III.** Total plasma FA in mol% of total plasma content of fatty acids in lipids at baseline and end of the study.

| **FA in mol% of total plasma FA** | **Mean/Median(95%CI or 90%percentil)** | **Mean/Median(95%CI or 90%percentil)** | ***p-val*** |
| --- | --- | --- | --- |
| ***SoC (n=15)*** | **Baseline** | **End** |  |
| Total SFA | 35.50 (33.82-37.19) | 35.27 (33.98-36.56) | 0.730 |
| C14:0 | 1.01(0.30-2.35) | 0.90 (0.28-2.207) | 0.208 |
| C16:0 | 27.13 (25.97-28.29) | 26.78 (25.86-27.71) | 0.492 |
| C18:0 | 7.25 (6.74-7.72) | 7.44 (6.86-8.030) | 0.239 |
| Total MUFA | 28.53 (27.25-29.81) | 28.16 (26.53-29.79) | 0.418 |
| C16:1 | 3.27 (2.80-3.74) | 2.97 (2.50-3.434) | 0.179 |
| C18:1n-9 | 25.26 (24.14-26.38) | 25.19 (23.70-26.69) | 0.870 |
| Total n-6 PUFA | 31.42 (29.72-33.12) | 32.86 (30.44-35.29) | 0.064 |
| C18:2n-6 | 24.19 (22.54-25.85) | 25.73 (23.10-28.36) | 0.113 |
| C20:3n-6 | 1.47 (0.00-1.82) | 1.22 (0.00-1.89) | 0.761 |
| C20:4n-6 | 5.50 (4.21-8.42) | 5.36 (4.16-8.57) | 0.454 |
| Total n-3 PUFA | 4.27 (2.44-9.10) | 3.74 (2.74-4.62) | 0.277 |
| C18:3n-3 | 0.68 (0.00-1.25) | 0.64 (0.00-1.07) | 0.078 |
| C20:5n-3 | 1.31 (0.20-4.00) | 0.87 (0.49-1.79) | 0.359 |
| C22:6n-3 | 2.12 (1.51-4.44) | 2.09 (1.70-2.56) | 0.454 |
| Sum of LA and ALA | 24.88 (23.23-26.53) | 26.32 (23.63-29.00) | 0.142 |
| n-3/n-6 | 0.14 (0.08-0.26) | 0.12 (0.08-0.16) | 0.055 |
| SCD-1(16:1/16:0) | 0.12 (0.10-0.13) | 0.11 (0.09-0.12) | 0.182 |
| D5 (20:4/20:3) | 3.51 (2.69-11.42) | 3.78 (2.84-8.92) | 0.831 |
|  |  |  |  |
| ***LCHF(n=15)*** |  |  |  |
| Total SFA | 35.24 (33.60-36.89) | 33.70 (32.67-34.72) | **0.026** |
| C14:0 | 0.87 (0.36-1.66) | 0.41 (0.00-1.13) | **0.002** |
| C16:0 | 26.72 (25.29-28.15) | 26.04 (25.11-26.97) | 0.169 |
| C18:0 | 7.61 (7.11-8.11) | 7.16 (6.56-7.76) | 0.074 |
| Total MUFA | 27.07 (25.03-29.10) | 26.28 (24.01-28.55) | 0.205 |
| C16:1 | 2.37 (1.91-2.83) | 1.71 (1.30-2.11) | **<0.001** |
| C18:1n-9 | 24.70 (22.91-26.48) | 24.57 (22.56-26.59) | 0.811 |
| Total n-6 PUFA | 33.57 (30.85-36.29) | 36.30 (33.63-38.98) | **0.001** |
| C18:2n-6 | 26.15 (23.48-28.82) | 28.30 (25.78-30.82) | **0.003** |
| C20:3n-6 | 1.44 (0.00-1.93) | 0.77 (0.00-1.34) | **<0.001** |
| C20:4n-6 | 6.04 (4.87-7.80) | 7.37 (4.94-9.61) | **0.012** |
| Total n-3 PUFA | 3.61 (2.10-8.58) | 3.02 (1.80-7.37) | 0.330 |
| C18:3n-3 | 0.68 (0.00-1.56) | 0.46 (0.00-1.19) | 0.068 |
| C20:5n-3 | 0.79 (0.42-3.30) | 0.51 (0.20-2.68) | 0.151 |
| C22:6n-3 | 2.03 (1.09-4.06) | 2.25 (1.32-3.76) | 0.524 |
| Sum of LA and ALA | 26.92 (24.19-29.65) | 28.81 (26.23-31.39) | **0.010** |
| n-3/n-6 | 0.10 (0.07-0.24) | 0.09 (0.05-0.21) | **0.005** |
| SCD-1(16:1/16:0) | 0.09 (0.07-0.10) | 0.07 (0.05-0.08) | **<0.001** |
| D5 (20:4/20:3) | 4.71 (2.96-6.99) | 6.81 (3.96-12.70) | **0.006** |
|  |  |  |  |
| ***5:2 (n=18)*** |  |  |  |
| Total SFA | 34.72 (33.63-35.80) | 33.65 (32.58-34.72) | 0.147 |
| C14:0 | 0.96 (0.61-1.71) | 0.51 (0.00-1.53) | **0.016** |
| C16:0 | 26.31 (25.51-27.11) | 26.18 (25.53-26.83) | 0.765 |
| C18:0 | 7.36 (6.92-7.81) | 6.88 (6.43-7.34) | 0.067 |
| Total MUFA | 28.71 (27.34-30.07) | 28.41 (26.51-30.31) | 0.596 |
| C16:1 | 2.48 (2.08-2.87) | 2.12 (1.72-2.53) | **0.006** |
| C18:1n-9 | 26.23 (24.79-27.67) | 26.29 (24.44-28.14) | 0.919 |
| Total n-6 PUFA | 32.61 (31.22-34.00) | 34.18 (32.34-36.03) | **0.027** |
| C18:2n-6 | 25.08 (23.28-26.87) | 25.97 (23.81-28.13) | 0.120 |
| C20:3n-6 | 1.52 (0.90-1.79) | 1.16 (0.00-1.93) | **0.030** |
| C20:4n-6 | 5.86 (3.94-8.64) | 6.80 (4.77-9.26) | **0.001** |
| Total n-3 PUFA | 3.95 (2.60-5.97) | 3.70 (1.89-5.91) | 0.284 |
| C18:3n-3 | 0.80 (0.39-1.45) | 0.55 (0.00-0.93) | **0.001** |
| C20:5n-3 | 0.88 (0.22-2.00) | 0.70 (0.00-1.80) | 0.060 |
| C22:6n-3 | 2.31 (1.18-3.21) | 2.51 (1.26-3.83) | 0.060 |
| Sum of LA and ALA | 25.88 (23.99-27.77) | 26.46 (24.28-28.64) | 0.270 |
| n-3/n-6 | 0.12 (0.07-0.18) | 0.10 (0.05-0.18) | 0.119 |
| SCD-1(16:1/16:0) | 0.09 (0.08-0.11) | 0.08 (0.07-0.09) | **0.003** |
| D5 (20:4/20:3) | 3.84 (3.02-6.86) | 4.99 (3.25-9.78) | **0.002** |

*For normally distributed values, the mean and CI 95% are reported and change between baseline and end of the study were tested with Student paired t-test. For non-normally distributed values, the median and 90% percentiles are reported and changes were tested for using Wilcoxon test to detect differences between baseline and end in the mol% of total fatty acids in plasma. LA=C18:2n-6, ALA=C18:3n-3.*

**Table IV.** Fatty acids as mol% of total plasma-FA at baseline.

| **Baseline**  **Plasma FA (mol%)** | **SoC (n=15)** | **LCHF (n=15)** | **5:2 (n=18)** | ***p-val*** |
| --- | --- | --- | --- | --- |
| **Total SFA** | 35.51 | 35.25 | 34.72 | 0.701 |
| C14:0 | 1.01 | 0.87 | 0.96 | 0.599 |
| C16:0 | 27.13 | 26.73 | 26.31 | 0.540 |
| C18:0 | 7.23 | 7.61 | 7.36 | 0.492 |
| **Total MUFA** | 28.53 | 27.07 | 28.71 | 0.245 |
| C16:1n-7 | 3.27 | 2.37 | 2.48 | 0.155 |
| C18:1n-9 | 25.27 | 24.70 | 26.24 | 0.281 |
| **Total n-6 PUFA** | 31.42 | 33.58 | 32.61 | 0.281 |
| C18:2n-6 | 24.20 | 26.16 | 25.08 | 0.387 |
| C20:3n-6 | 1.47 | 1.44 | 1.52 | 0.393 |
| C20:4n-6 | 5.50 | 6.04 | 5.86 | 0.807 |
| **Total n-3 PUFA** | 4.27 | 3.61 | 3.95 | 0.300 |
| C18:3n-3 | 0.68 | 0.68 | 0.80 | 0.650 |
| C20:5n-3 | 1.31 | 0.79 | 0.88 | 0.290 |
| C22:6n-3 | 2.12 | 2.02 | 2.31 | 0.732 |
| n-3/n-6 | 0.14 | 0.10 | 0.12 | 0.184 |
| Sum of LA and ALA | 24.88 | 26.92 | 25.88 | 0.379 |
| SCD-1(16:1/16:0) | 0.12 | 0.09 | 0.09^#^ | **0.004** |
| D5 (20:4/20:3) | 3.51 | 4.71 | 3.84 | 0.902 |

*Normally distributed values were tested for differences between groups using one-way ANOVA, or for non-normally distributed values, Kruskal Wallice, was used. * <0,05 for SoC vs LCHF, € <0,05 for SoC vs 5:2 and # < 0,05 LCHF vs 5:2 for the post-hoc test.*

**
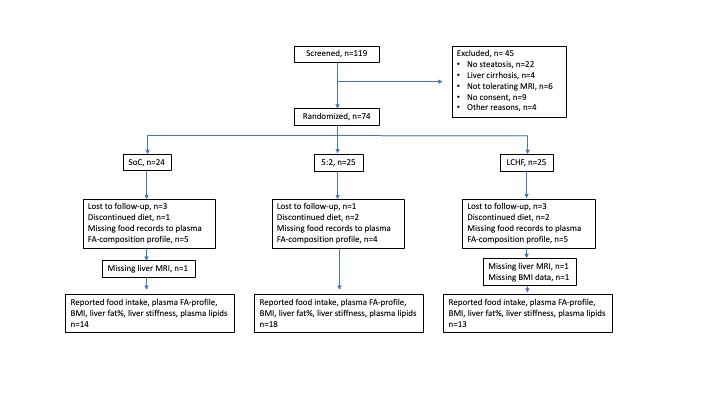
**

**Supplementary figure 1.** Outline of the inclusion of participants for post-hoc analysis

**
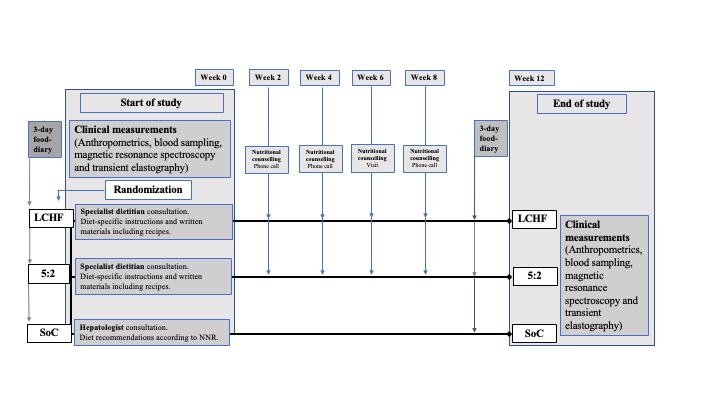
**

**Supplementary figure 2.** Outline of the study protocol
